# Olaparib maintenance therapy for recurrent adult granulosa cell tumors: A case report

**DOI:** 10.1111/jog.70078

**Published:** 2025-09-19

**Authors:** Haruna Tsukahara, Ichiro Onoyama, Kazuhisa Hachisuga, Hiroshi Yagi, Hideaki Yahata, Kiyoko Kato

**Affiliations:** ^1^ Department of Obstetrics and Gynecology, Graduate School of Medical Science Kyushu University Fukuoka Japan

**Keywords:** adult granulosa cell tumor, homologous recombination deficiency, olaparib maintenance therapy

## Abstract

Poly (ADP‐ribose) polymerase inhibitors (PARPi) are now available for advanced or recurrent epithelial ovarian cancer after platinum‐based chemotherapy; however, there is little known concerning the use of PARPi for adult granulosa cell tumors (AGCT). A 49‐year‐old woman, who previously had primary debulking surgery and a second surgery for a first intraperitoneal recurrence, received a surgery for a second intraperitoneal recurrence of AGCT. Although she received paclitaxel–carboplatin combination chemotherapy and a combination of bleomycin, etoposide, and cisplatin after the surgeries for the first and the second recurrences, respectively, the risk of a third recurrence was considered very high due to microscopic peritoneal dissemination. Olaparib administration was initiated based on a *FANCA* mutation, which results in homologous recombination deficiency. The patient has been recurrence‐free for over 4 years since the initiation of olaparib. This case highlights the possibility of using olaparib for AGCT with a homologous recombination gene mutation.

## INTRODUCTION

Adult granulosa cell tumors (AGCT), one of the sex‐cord stromal tumors with low malignant potential, account for 2%–5% of ovarian malignancies. Most AGCTs are limited to the ovary at the time of diagnosis, with a relatively favorable prognosis; however, AGCTs recur in 20%–25% of patients after the initial surgery.^1^ Unpredictable recurrence after a long interval is one of the clinical characteristics of AGCT. Platinum‐based chemotherapy combined with cytoreductive surgery is used for recurrent AGCT, although there is no standardized therapy at present. Paclitaxel–carboplatin combination chemotherapy (TC) is sometimes used for recurrent AGCT, and another option is a combination of bleomycin, etoposide, and cisplatin (BEP).^2^ However, the response rate for these chemotherapies is insufficient, and their effects are still controversial. Accordingly, the development of treatment for recurrent AGCT and prevention of further recurrence are keys to managing patients with AGCT.

Recently, poly (ADP‐ribose) polymerase (PARP) inhibitors (PARPi) have emerged as a maintenance therapy for ovarian cancer. PARPi have been shown to be very effective for preventing recurrence, especially for tumors with homologous recombination deficiency (HRD). PARPi are available for advanced or recurrent ovarian cancer; however, they are currently only used for epithelial ovarian cancers. To the best of our knowledge, there have been no reports of PARPi used for recurrent AGCT with *FANC* gene mutation.

Here, we describe a patient with recurrent ACGT maintained with the PARPi olaparib as a case report. The tumor has a mutation in *FANCA*, one of the homologous recombination repair genes, which impairs the FANC–BRCA pathway.

## CASE REPORT

**FIGURE 1:**
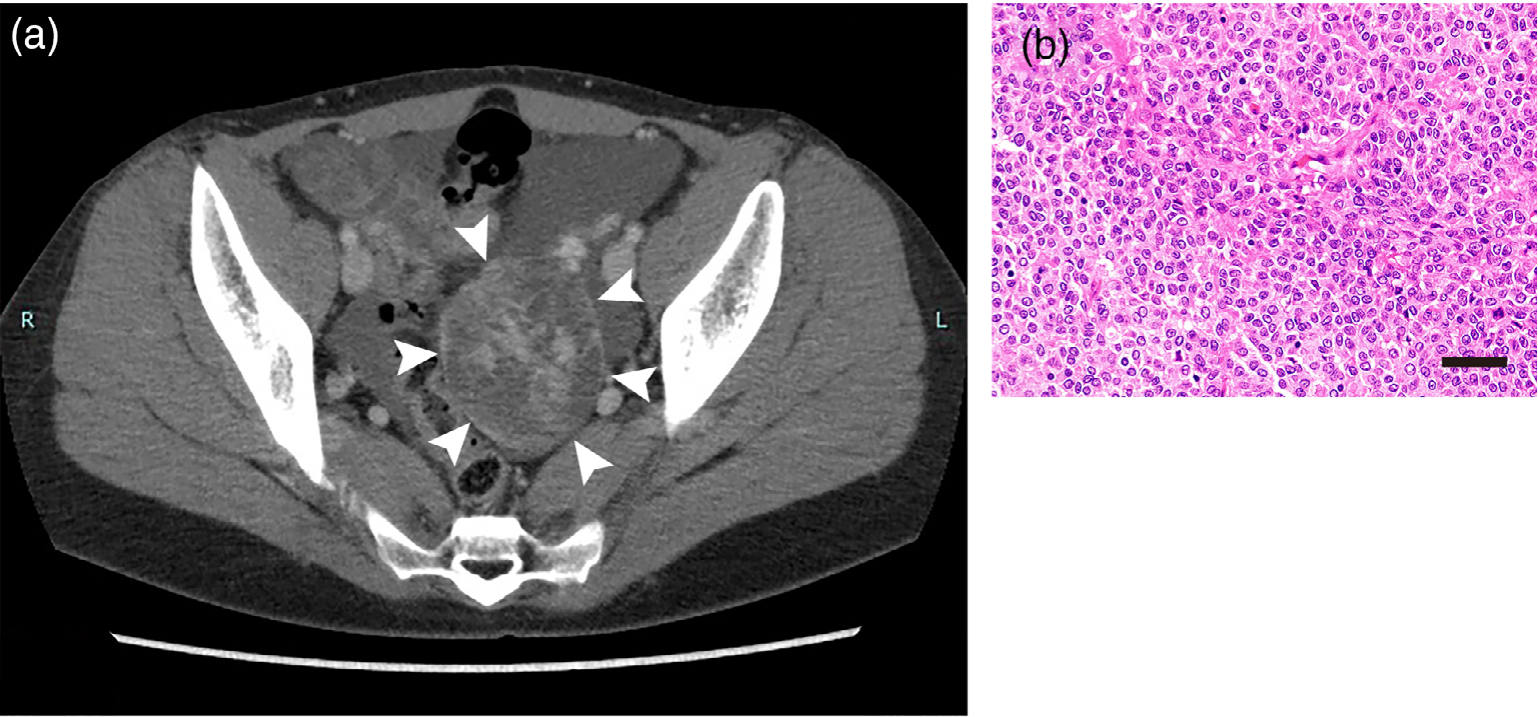
The first recurrence 8 years after laparoscopic left ovarian cystectomy. (a) Computed tomography shows a recurrent pelvic mass with irregular enhancement in the left side of the rectum (arrow heads). (b) Hematoxylin and eosin staining of one of the resected pelvic tumors. Left adnexa tumor was composed of a proliferation of relatively uniform round‐to‐oval tumor cells with scant or eosinophilic cytoplasm, arranged in diffuse and microfollicular diffuse sheets with an insular pattern. Grooved nuclei are observed. Mitotic figure is seen (8/10HPFs). Scale bar: 40 μm.

**FIGURE 2:**
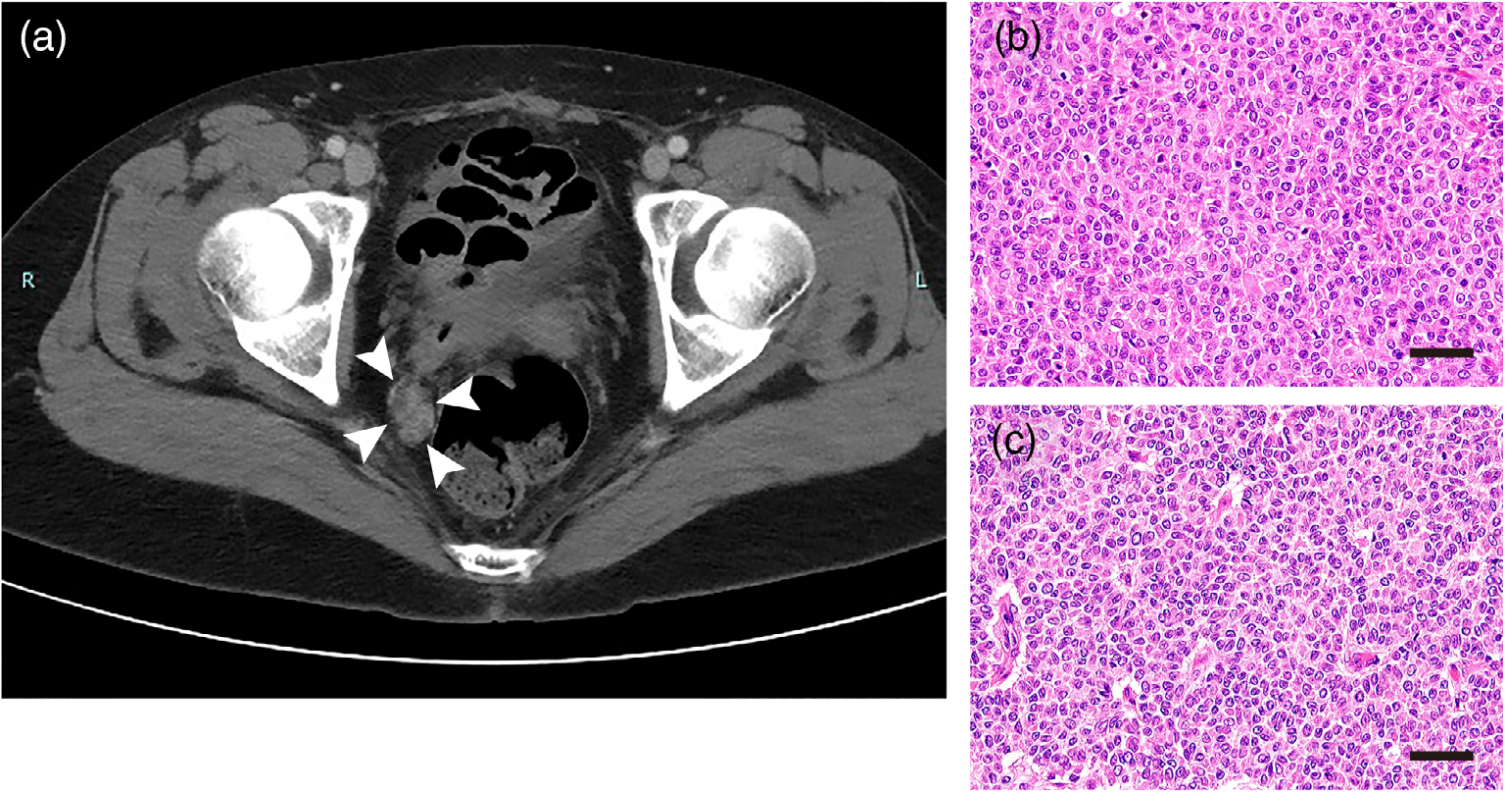
The second recurrence 2 years and 1 month after six cycles of TC chemotherapy. (a) Computed tomography shows the recurrent pelvic mass in the right side of the rectum (arrow heads). (b) Hematoxylin and eosin staining of one of the resected pelvic tumors. Similar findings in the first recurrence are seen. (c) Hematoxylin and eosin staining of omentum resected in the second recurrence. The omental tissue showed small nodular proliferation of round‐to‐oval tumor cells. Scale bar: 40 μm. TC, paclitaxel–carboplatin combination chemotherapy.

The patient is a 49‐year‐old woman, gravida 3, para 1, with an unremarkable medical and family history. Informed consent was obtained from the patient. She received a laparoscopic left ovarian cystectomy when she was 34 years old. Pathological examination revealed AGCT; however, she refused a staging laparotomy at that time. International Federation of Gynecology and Obstetrics (FIGO) stage was considered stage IC1. She was found to have relapsed 8 years later with three pelvic masses of 71, 37, and 27 mm in diameter as detected by computed tomography (Figure 1a), and she was admitted to our hospital for complete removal of the three pelvic masses. Here estradiol levels were not a reliable marker because they were varied. Inhibin and anti‐mullerian hormone had not been examined because the health insurance does not cover them in Japan. She received an abdominal hysterectomy, bilateral salpingo‐oophorectomy, partial omentectomy, appendectomy, and partial rectal resection. Pathological examination showed recurrent AGCT of the left ovary, along with disseminations to the omentum and rectal serosa (Figure 1b). Ascitic fluid cytology was negative. Six cycles of TC were performed after the second surgery, with no sign of recurrence. Two years and one month after the six cycles of TC chemotherapy, she was found to have relapsed, with multiple pelvic masses of approximately 20 mm in diameter (Figure 2a). Cytoreduction surgery was performed, and three disseminations were removed (Figure 2b). There were no gross lesions remaining in the intra‐abdominal cavity; however, AGCT cells were histopathologically recognized in macroscopically normal omentum (Figure 2c). Ascitic fluid cytology was negative. The patient then received four cycles of BEP chemotherapy.

Cancer genome profiling (CGP) using a specimen from the second recurrence was performed with Foundation One®, and revealed mutations in three genes: *FANCA* (M1L), *TERT* promoter (124C>T), and *FOXL2*. The variant allele frequency (VAF) of the *FANCA* mutation is 48.19%. *FANCA* is one of the homologous recombination repair genes, in which dysfunction leads to HRD.^3^, ^4^ The second recurrence was considered a platinum‐sensitive relapse as there was no sign of recurrence after four cycles of BEP chemotherapy. Consequently, oral administration of olaparib maintenance therapy (600 mg/day) was initiated to prevent a third relapse. More than 4 years have passed with olaparib maintenance therapy, and there is no sign of a third recurrence. No adverse events were reported during maintenance therapy.

## DISCUSSION

This case describes the use of olaparib maintenance therapy for recurrent AGCT. The prognosis for AGCT is generally favorable; however, AGCT is known to recur after a long interval. The median time to relapse after initial treatment is 5 years, and a recurrence as long as 40 years after treatment has also been reported.^5^ Given that AGCT shows such a slow growth and low malignancy potential, it may be possible that AGCT shows a relatively low response to chemotherapy. Platinum‐based chemotherapy, such as the TC and BEP regimens, is generally indicated for recurrence after cytoreductive surgery. The response rate of TC is 42%, and that of BEP is 37%.^6^, ^7^ Therefore, there is an urgent need to establish maintenance therapy as well as new chemotherapy regimens for AGCT.

In the case presented here, TC and BEP regimens were performed after cytoreduction surgery for the first and second recurrences, respectively. Risk of a third recurrence was considered very high, because microscopic intraperitoneal disseminations to the omentum were observed in the second recurrence as well as in the first recurrence. Thus, olaparib maintenance therapy was initiated after four cycles of BEP based on the determination of an M1L mutation in *FANCA* by CGP. CGP also detected a *FOXL2* mutation, which genetically supports the AGCT diagnosis.^8^

*FANCA* is one of the causative genes of Fanconi anemia and is a component of the FANC core complex. The FANC core complex mono‐ubiquitylates the FACND2‐FANCI complex, which induces homologous recombination repair of damaged DNA together with BRCA1 and BRCA2 proteins.^3^, ^4^ Thus, *FANCA* gene mutations lead to homologous recombination repair deficiency as well as *BRCA* gene mutations. The *FANCA* mutation (M1L) found in this case would result in a lack of FANCA protein expression, leading to HRD.^9^ This provided a rationale for olaparib maintenance therapy.

PARPi are now among the key drugs in ovarian cancer management. They provide a great benefit in patients with HRD tumors and those with platinum‐sensitive relapse. Many clinical trials of PARPi have been reported, including SOLO‐1, SOLO‐2, study 19, PRIMA, and NOVA.^10^, ^11^, ^12^, ^13^, ^14^ However, only epithelial ovarian cancers have been included in these clinical trials, with no inclusion of patients with AGCT. It is reported that potential pathogenic variants in a DNA repair‐related gene (*ATM*, *BRCA1*, *BRCA2*, *CHEK2*, *PALB2*, *PMS2*, *RAD51C*, or *RAD51D*) were found in 6.6% of 516 *FOXL2*‐mutated AGCT patients. One patient with an *ATM* mutation received olaparib, but the disease progressed after 13 months.^15^ More than 4 years have passed with no evidence of disease since the introduction of olaparib in our case, but it is difficult to evaluate the efficacy of olaparib alone as BEP chemotherapy was also effective in suppressing the disease. Although it might be possible that PARPi is effective in this case because the VAF of the *FANCA* mutation is high, this patient should be followed for a longer period.

Given that mutation in *FOXL2* is commonly seen in AGCT, FOXL2 is considered as a candidate for targeted therapy.^2^ However, no targeted therapy is currently available for AGCT. Although more data is needed to investigate the frequency of mutations in homologous recombination repair genes and the efficacy of PARPi in AGCT, this case highlights the possibility of the use of PARPi for recurrent AGCT.

Recurrent AGCT is very difficult to cure; in the presence of inoperable recurrence, there are no treatment options resulting in a durable response. One of the main reasons may be a lack of effective chemotherapy or targeted therapy against AGCT. Although the present case study is limited to the description of a single patient, this case report may lead to further study on PARPi and the development of new targeted therapies for AGCT.

## AUTHOR CONTRIBUTIONS

**Haruna Tsukahara:** Writing – original draft; writing – review and editing; conceptualization; data curation; conceptualization; data curation; writing – original draft; writing – review and editing; writing – review and editing; data curation; writing – review and editing; supervision; writing – review and editing; supervision; writing – review and editing; supervision. **Ichiro Onoyama:** Conceptualization; data curation; writing – original draft; writing – review and editing. **Kazuhisa Hachisuga:** Writing – review and editing; data curation. **Hiroshi Yagi:** Writing – review and editing; supervision. **Hideaki Yahata:** Writing – review and editing; supervision. **Kiyoko Kato:** Writing – review and editing; supervision.

## CONFLICT OF INTEREST STATEMENT

The authors declare that they have no known competing financial interests or personal relationships that could have appeared to influence the work reported in this paper. Dr. Kazuhisa Hachisuga and Dr. Hideaki Yahata are Editorial Board members of this submitted JOGR Journal and co‐authors of this article. To minimize bias, they were excluded from all editorial decision‐making related to the acceptance of this article for publication.

## ETHICS STATEMENT

The case report does not need to go through an ethics committee if the patient's data is not recognizable.

## CONSENT

Written informed consent was obtained from the patient for the publication of her de‐identified data and images.

## Data Availability

Data sharing not applicable to this article as no datasets were generated or analysed during the current study.
